# Bone Marrow Mesenchymal Stem Cell-Derived Exosomes Accelerate Functional Recovery After Spinal Cord Injury by Promoting the Phagocytosis of Macrophages to Clean Myelin Debris

**DOI:** 10.3389/fcell.2021.772205

**Published:** 2021-11-08

**Authors:** Xiaolong Sheng, Jinyun Zhao, Miao Li, Yan Xu, Yi Zhou, Jiaqi Xu, Rundong He, Hongbin Lu, Tianding Wu, Chunyue Duan, Yong Cao, Jianzhong Hu

**Affiliations:** ^1^Department of Spine Surgery and Orthopaedics, Xiangya Hospital, Central South University, Changsha, China; ^2^Key Laboratory of Organ Injury, Aging and Regenerative Medicine of Hunan Province, Changsha, China; ^3^Hunan Engineering Research Center of Sports and Health, Changsha, China; ^4^National Clinical Research Center for Geriatric Disorders, Xiangya Hospital, Central South University, Changsha, China; ^5^Department of Orthopedics, Hunan Children’s Hospital, Changsha, China; ^6^Department of Sports Medicine, Xiangya Hospital, Central South University, Changsha, China; ^7^Department of Pain, Institute of Pain Medicine, Third Xiangya Hospital of Central South University, Changsha, China

**Keywords:** BMSC derived exosomes, macrophage, phagocytosis, myelin debris, spinal cord injury

## Abstract

Macrophage phagocytosis contributes predominantly to processing central nervous system (CNS) debris and further facilitates neurological function restoration after CNS injury. The aims of this study were to evaluate the effect of bone marrow mesenchymal stem cells (BMSC)-derived exosomes (BMSC-Exos) on the phagocytic capability of macrophages to clear myelin debris and to investigate the underlying molecular mechanism during the spinal cord injury (SCI) process. This work reveals that monocyte-derived macrophages (MDMs) infiltrating into the SCI site could efficiently engulf myelin debris and process phagocytic material. However, the phagocytic ability of macrophages to clear tissue debris is compromised after SCI. The administration of BMSC-Exos as an approach for SCI treatment could rescue macrophage normal function by improving the phagocytic capability of myelin debris internalization, which is beneficial for SCI repair, as evidenced by better axon regrowth and increased hindlimb locomotor functional recovery in a rodent model. Examination of macrophage treatment with BMSC-Exos revealed that BMSC-Exos could promote the capacity of macrophages to phagocytose myelin debris *in vitro* and could create a regenerative microenvironment for axon regrowth. In addition, we confirmed that BMSC-Exo treatment resulted in improved phagocytosis of engulfed myelin debris by promoting the expression of macrophage receptor with collagenous structure (MARCO) in macrophages. The inhibition of MARCO with PolyG (a MARCO antagonist) impaired the effect of BMSC-Exos on the phagocytic capacity of macrophages and resulted in compromised myelin clearance at the lesion site, leading to further tissue damage and impaired functional healing after SCI. In conclusion, these data indicated that targeting the phagocytic ability of macrophages may have therapeutic potential for the improvement in functional healing after SCI. The administration of BMSC-Exos as a cell-free immune therapy strategy has wide application prospects for SCI treatment.

## Introduction

Spinal cord injury (SCI) is a devastating event that may result in permanent damage to sensation and motor functions below the injured site of the spinal cord (Huang et al., 2020a; Li Y. et al., 2020; Zrzavy et al., 2021). SCI provokes an inflammatory response after primary injury consisting of activated resident and infiltrating immune cells (Sefiani and Geoffroy, 2021). A remarkable feature of the inflammatory processes in SCI is the infiltration of monocyte-derived macrophages (MDMs) from the circulation to the lesion site (David et al., 2018). The initial injury triggers inflammation, contributes to oligodendrocyte and neuronal cell death, and causes the accumulation of excessive myelin and cellular debris at the lesion site of the spinal cord (Gensel and Zhang, 2015). It has been reported that myelin debris inhibits axonal regeneration and impedes neurite outgrowth, and myelin debris can also act as an inflammatory stimulus (Zhou T. et al., 2019; Liu Y. et al., 2021). Successful neurological functional recovery after central nervous system (CNS) injury depends on the clearance of myelin debris. Damaged myelin debris is engulfed by MDMs if the blood–brain barrier is disrupted, which is essential for creating a pro-regenerative environment (Wang et al., 2015).

Following SCI, various pathological changes occur, including inflammation, proliferation, and remodeling phases. MDMs are the key player in orchestrating the sequential transition among these phases by secreting multiple signaling molecules (Evans et al., 2014). MDMs are also considered to be the most important cellular cues in the clearance of myelin debris (Van Broeckhoven et al., 2021). However, the phagocytic ability of MDMs could be compromised after SCI (Gensel and Zhang, 2015; Guo et al., 2016; Van Broeckhoven et al., 2021). If the decreased myelin clearance persists in the lesion site, it may inhibit the remyelination of axons, further leading to the deterioration of motor functional recovery post-SCI (Zhou Q. et al., 2019).

Indeed, clinical studies have revealed that myelin debris is not cleared from the injured spinal cord until years after injury (Kopper and Gensel, 2018). Accumulating evidence indicates that the sustained presence of myelin debris in the injured spinal cord not only inhibits axon regeneration but also acts as a potent inflammatory stimulus that may aggravate further tissue damage to the spinal cord (Li F. et al., 2020; Wang et al., 2020; Wan et al., 2021). Thus, it is clinically important to enhance the phagocytic capacity of macrophages and promote their uptake of damaged myelin debris, which may exert beneficial effects on functional recovery after SCI.

Many approaches have been employed to target the macrophage phagocytic ability to improve neurological functional restoration after SCI. For instance, embryonic stem cells (ESCs) have been reported to have the ability to enhance the phagocytic activity of macrophages (Guo et al., 2016). Most recent studies have focused on the application of exosomes derived from bone morrow mesenchymal stem cells (BMSCs) for SCI treatment (Liu W. Z. et al., 2021). It has been demonstrated that the transplantation of BMSCs leads to functional repair of CNS trauma mainly through the secretion of exosomes rather than differentiation of BMSCs (Huang et al., 2017, 2020c; Liu W. Z. et al., 2021).

Exosomes can be easily isolated and can cross the spinal cord–blood barrier and reach the injured site in the CNS system (Yuan et al., 2019). Exosomes carry a large number of molecules, such as proteins and various nucleic acids [mRNAs, miRNAs, and other long non-coding RNAs (lncRNAs)] (Feng et al., 2021). At present, a clinical trial of mesenchymal stem cell (MSC)-derived exosomes for the treatment of SCI has been conducted (Nikfarjam et al., 2020; Lee et al., 2021). It has also been revealed that MSC-exosomes could infiltrate into the local injured site and alter the phenotypic transformation of macrophages from M1 to M2 (Lankford et al., 2018). However, the effect of MSC-derived exosomes on the phagocytosis activity of macrophages has not been demonstrated. Therefore, these prior findings prompted us to clarify whether BMSC-derived exosomes (BMSC-Exos) could regulate macrophage phagocytic function in an SCI model.

In our current study, we evaluated the therapeutic effects of BMSC-Exos in the treatment of SCI and explored the underlying mechanism. We identified that BMSC-Exo treatment could promote neurological functional restoration after SCI by enhancing macrophage phagocytosis capacity and promoting macrophage engulfment of myelin debris through upregulation of macrophage receptor with collagenous structure (MARCO) in macrophages.

## Materials and Methods

### Mice

Female C57BL/6 mice (8–10 weeks old) were used for this study and were maintained in a pathogen-free animal facility at Central South University. All animal protocols and experimental procedures were approved by the Animal Care and Use Committee of Central South University. The mice were housed in the laboratory Animal Unit on a 12-h day/night cycle, with *ad libitum* access to food and water, and were allowed to acclimatize for 1 week before the animal model was generated.

### Isolation and Characterization of Mouse Bone Marrow Mesenchymal Stem Cell-Derived Exosomes

Mouse BMSCs were purchased from the Cell Bank (Type Culture Collection, Chinese Academy of Sciences, Shanghai, China) and cultured in 96-well plates with 100 μl of Dulbecco’s modified Eagle’s medium (DMEM)/F12 complete medium with 10% fetal bovine serum (FBS) supplemented with 1% L-glutamine and 1% antibiotic-anti-mycotic solution (all from Gibco, Thermo Fisher, Waltham, MA, United States). After the cells reached 80% confluence, serum and exosome-free culture medium was added, after 48-h cell culture, and the supernatant was collected. The exosomes were isolated from the supernatant by using an optima L-100 XP Ultracentrifuge (Beckman Coulter, Brea, CA, United States) as previously reported (Kalluri and LeBleu, 2020). Then, the isolated exosomes were resuspended in 0.01 M of phosphate-buffered saline (PBS) at a concentration of 500 μg/μl and used immediately for downstream experiments. Exosome morphology was detected using transmission electron microscopy (TEM; Philips-Tecnal, Eindhoven, Netherlands). Nanoparticle tracking analysis (NTA) by Nanosight (LM10-HS, Malvern, Westborough, MA, United States) was used to measure the exosome concentration and size distribution. The protein content in exosomes and exosomes-specific surface markers, such as anti-CD63 (Abcam, 1/250, United States), anti-CD9 (Abcam, 1/250, United States), and anti-TSG101 (System Biosciences, 1/1,000, CA, United States), were examined using a Pierce BCA Protein Assay Kit (Thermo Fisher Scientific, Rockford, IL, United States) and Western blotting according to previous reports (Huang et al., 2020b).

### Mouse Bone Marrow-Derived Macrophage Preparation

Mouse bone marrow cells were isolated from C57BL/6 mouse femoral shafts by flushing the femur marrow cavity with DMEM supplemented with 1% FBS according to previously described methods (Wang et al., 2012). Red blood cell lysate was added to remove the red blood cells and finally to obtain bone marrow cells. Sorted monocytes or bone marrow cells were treated with 20 ng/ml of recombinant human or mouse granulocyte macrophage colony-stimulating factor (GM-CSF) to induce macrophages.

### Cortical Neuron Axonal Growth Assay

Primary cortical neurons were isolated from the cerebral cortex of E16 embryonic C57BL/6 mouse pups according to previously described methods (Kim and Magrane, 2011). The cortices were dissected out. After enzyme dissection, the extracted cortical neurons were counted and plated on sterilized cover glass in six-well plates in serum-free neurobasal medium supplemented with appropriate growth supplements (B27 and N2, Life Technologies, Carlsbad, CA, United States) to remove astrocytes, oligodendrocytes and microglia. Primary cortical neurons were cultured with different concentrations of myelin debris for 48 h. Cells were fixed and stained with anti-β-tubulin III (1/400, Abcam). Stained images were visualized using a fluorescence microscope (Carl Zeiss Axio Observer Z1, Oberkochen, Germany), and axon length was measured using ImageJ software (National Institutes of Health, Bethesda, MD, United States) in a blinded manner.

### Tracking of Administered Bone Marrow Mesenchymal Stem Cell-Derived Exosomes

Bone marrow mesenchymal stem cells -Exos were fluorescently labeled with PKH67 dye (Sigma-Aldrich Corp., St. Louis, MO, United States) as previously described (Ohno et al., 2013). Briefly, 2.5 μl of PKH67 (100 μg/ml) was added to 50 μl of BMSC-Exos (1 mg/ml) for 1 h in dark spin columns (MW3000, Invitrogen, Carlsbad, CA, United States). Spin columns in the collection tubes were centrifuged using an AllegraX-15R (Beckman, United States), and the columns were placed in 1.5-ml tubes and centrifuged at 750 × *g* to collect labeled BMSC-Exos. To track BMSC-Exo uptake by bone marrow-derived macrophages (BMDMs) *in vitro*, 10 μg/ml of PKH67-labeled exosomes was added to the culture medium of BMDMs and incubated for 24 h. Cells were then washed with PBS and fixed with 4% paraformaldehyde (PFA; pH = 7.4) for immunostaining, and 4′,6-diamidino-2-phenylindole (DAPI; 0.5 μg/ml; Invitrogen, Carlsbad, CA, United States) was used for nuclear visualization. F4/80 (1/200, eBioscience, San Diego, CA, United States) was used for macrophage staining. The uptake of PKH67-labeled exosomes by BMDMs was then viewed and photographed using a fluorescence microscope (Carl Zeiss Axio Observer Z1, Oberkochen, Germany).

For tracking BMSC-Exos *in vivo* and for maintaining sustained exosomes release at the injured site, we labeled exosomes with 1,1-dioctadecyl-3,3,3,3-tetramethylindotricarbocyanine iodide (DiR) (100 μg/ml, 2 μl, Invitrogen) and mixed them with hydrogel before implantation according to the method used in our previous study using a Xenogen IVIS Imaging System (Caliper Life Sciences, Waltham, MA, United States) (Cao et al., 2021). PKH67-labeled BMSC-Exos were administrated after SCI, and the spinal section was fluorescently costained with F4/80 *in vivo* to revealed the uptake exosomes by macrophage cells.

### Spinal Cord Contusion Model in Mice

Thoracic spinal cord contusion injuries at the T10 vertebrae were performed on female mice as previously described (Cao et al., 2017). Briefly, mice were anesthetized with ketamine (80 mg/kg) and xylazine (10 mg/kg) through intraperitoneal injection. Then, a laminectomy was performed to expose the spinal cord at T10, and contusion injury was generated using an NYU impactor with a 5-g rod dropped from a height of 6 mm from the spinal cord surface. Mice in the sham groups were subjected to laminectomy without contusion injury. Spinal cord contusion mice were randomly and blindly divided into the PBS and BMSC-Exo treatment groups. After the operation, all mice received subcutaneous injections of meloxicam (1 mg/kg) as an analgesic.

### Preparation of Myelin Debris and Myelin Debris Uptake Assay

Myelin debris was isolated from the brains of 2-month-old mice by sucrose density gradient centrifugation as described previously (Grajchen et al., 2020). Myelin debris was added to the cultured BMDMs at a final concentration of 1 mg/ml. Non-ingested myelin debris was washed away from the cell surface. After removal of non-ingested myelin debris, cells were fixed with 4% PFA, followed by anti-mouse myelin basic protein (MBP) antibody (1/400, Abcam) immunostaining for myelin debris engulfment detection. Images were captured with a fluorescence microscope (Carl Zeiss Axio Observer Z1, Oberkochen, Germany). Oil Red O (ORO) staining was applied to visualize macrophages phagocytosing myelin debris *in vitro*. Stained samples were captured with a Leica light microscope (*n* = at least three independent biological replicates).

### Bone Marrow Mesenchymal Stem Cell-Derived Exosomes Administration

For *in vitro* experiments, macrophages were treated with 10 μg/ml of BMSC-Exos for 24 h to ensure incorporation of the exosomes. Then, the exosome-treated macrophages were incubated with myelin debris to evaluate the phagocytic capacity change (*n* = at least three independent biological replicates). For *in vivo* SCI animals, 200 μl of BMSC-Exos at a concentration of 1 mg/ml was mixed with hydrogel and then injected locally immediately on the surface of the injured spinal cord as previously described post-SCI (Cao et al., 2021). For the naïve control groups of mice, an equivalent volume of PBS was applied using the same approach (*n* = 5/group for each time point). After administration of different interventions, mice were anesthetized and perfused at the corresponding time point post-SCI, and then the spinal cords were collected for subsequent experiments.

### The Inhibition of Macrophage Receptor With Collagenous Structure Both *in vivo* and *in vitro*

To explore the underlying mechanism by which exosomes regulate phagocytic processes by macrophages, the phagocytic receptor MARCO antagonist PolyG (Sigma) was applied to both *in vitro* cell culture and *in vivo* animal models.

For the SCI model, the mice were randomly divided into BMSC-Exo treatment groups and BMSC-Exos plus PolyG (2 mg/kg body weight, Sigma) treatment groups. The exosomes administered in combination with PolyG were mixed with hydrogel and then injected locally on the surface of the injured spinal cord according to our previously described method (Cao et al., 2021). For the cell culture study, macrophage cells were precultured with 200 μg/ml of PolyG (Sigma) for 30 min. Then, myelin debris uptake by the macrophages was evaluated with or without added BMSC-Exos.

### Myelin Debris-Treated Bone Marrow-Derived Macrophages and Cortical Neuron Coculture Assay

For BMDMs and cortical neuron coculture, we used a Transwell system in which cortical neurons were seeded in the lower chamber and BMDMs precultured with myelin debris were seeded in the upper chamber. The myelin debris-treated BMDMs in the upper chamber were divided into two groups, namely, with or without BMSC-Exos administration. After 48 h of coculture, the cortical neuron cells in the lower chamber were fixed and stained with anti-β-tubulin III (1:400, Abcam) for axon visualization. The axon length was measured using ImageJ software (National Institutes of Health, United States).

### Behavioral and Neurological Functional Recovery Analyses

Functional recovery of the hindlimb motor function of SCI mice was assessed preinjury and on days 1, 3, 5, 7, 10, 14, 21, 28, 42, and 56 post-SCI after the administration of BMSC-Exos using the open-field Basso Mouse Scale (BMS) score evaluation system as previously described (Basso et al., 2006).

The BMS scores ranged from 0 (complete paralysis) to 9 (normal movement function of the hindlimbs). The 11-point BMS subscore, including the frequency of plantar stepping, coordination, paw position, trunk stability, and tail position, was also evaluated and used to supplement the main scale. The average BMS scores and subscores for both the left and right hindlimbs were recorded by two well-trained investigators who were blinded to the experimental design. Electromyography was used to assess the motor evoked potentials (MEPs) of each mouse in different treatment groups at day 56 after SCI following previously described methods (Cao et al., 2021). Briefly, mice were anesthetized with ketamine (80 mg/kg) and xylazine (10 mg/kg) through intraperitoneal injection. The positive electrode was placed on the skull surface of the motor area of the cerebral cortex, and the negative electrode was placed on the skull 0.5 cm lateral to the positive electrode. The recording electrode was inserted into the left or right gastrocnemius muscle of the hindlimbs. A reference electrode was then inserted at the distal tendon of the hindlimb muscle, and the ground electrode was placed under the skin. A 4-mA single square wave (2 Hz) was used to stimulate the motor area of the cerebral cortex through the skull with a duration of 0.2 ms. The amplitude of the hindlimbs was calculated from the initiation point of the first response wave to its highest peak (Nardone et al., 2015).

### Histological Assessment of the Injured Spinal Cord

In addition to the neurological functional analysis, after the animals were transcardially perfused with 4% PFA, we performed a histological assessment of spinal cord slices (8-μm thickness) 1,000 μm rostrally from the injured epicenter at 56 days post-SCI using hematoxylin and eosin (H&E) staining. The lesion sites in H&E-stained images were carefully examined using ImageJ software (National Institutes of Health, United States).

### Immunofluorescence Analysis

For immunofluorescence analysis, serial sections of 10-μm thickness from the spinal cord containing the lesion site were harvested at 7 days post-SCI and cut along the sagittal plane. Immunostaining analysis was performed according to standard protocols. Briefly, sagittal spinal cord sections were permeabilized with 0.3% Triton X-100 in PBS and blocked with 5% bovine serum albumin (BSA) in PBS. Following blocking, sections were incubated with primary antibodies, including anti-F4/80 (1/400, Abcam), anti-β-tubulin III (1/200, Abcam), and anti-MBP antibodies (1/400, Abcam), overnight. After being rinsed in PBS, the sections were incubated with species-appropriate secondary antisera conjugated with Alexa Fluor 568 (1/400, Abcam) or Alexa Fluor 488 (1/400, Abcam), mounted on slides, and then cover slipped with Vectashield (Vector Laboratories Inc., Burlingame, CA, United States).

Images were captured by a fluorescence microscope. Positive cells in at least three randomly selected visual fields per section were quantified using ImageJ software. The area and distribution density of never fibers were examined in region of interest (ROI) selected at the midline sagittal area of the spinal cord using ImageJ software.

For the quantification of myelin debris uptake by macrophages *in vivo*, F4/80-positive cells with intracellular MBP laden were considered myelin-containing macrophages and were randomly selected from three visual views per section for careful analysis using ImageJ software. In some images, the x–y, x–z, and y–z views were obtained and used to visualize the presence of myelin debris within F4/80-positive macrophage cells.

For analysis of the expression of MARCO in macrophages after administration of exosomes, we costained macrophage with anti-MARCO (1/250 for culture cells, 1/500 for spinal cord slice staining, Abcam) and anti-F4/80 (1/200 for culture cells, 1/400 for spinal cord slice staining, Abcam) in both the injured spinal cord section and culture cells. The area and fluorescence density of MARCO staining in F4/80-positive cells in three randomly selected fields was quantitatively analyzed using ImageJ software.

### RNA Isolation and Quantitative Real-Time PCR

RNA was extracted using TRIzol^®^ reagent (Invitrogen). cDNA was reverse transcribed from 1 μg of RNA using a qScript Flex cDNA Synthesis Kit (Quanta Biosciences, Beverly, MA, United States). A 20-μl reaction system was prepared for quantitative RT-PCR using SYBR Green PCR master mix (Applied Biosystems, Foster City, CA, United States) on an ABI 7900 fast real-time PCR system (Applied Biosystems). The expression levels of target genes were normalized to internal controls (GAPDH) and calculated using the 2^–ΔΔCT^ method. A full list of the primers used is provided in Table 1.

**TABLE 1:** List of qPCR primers used to validate genes related to phagocytizing myelin debris.

**Gene**	**Forward primer (5′–3′)**	**Reverse primer (5′–3′)**
*GAPDH*	AGCAAGGACACTGAGCAAGA	GGGGTCTGGGATGGAAATTGT
*CD36*	TTTGGAGTGGTAGTAAAAAGGGC	TGACATCAGGGACTCAGAGTAG
*SR-AI/II*	TGGAGGAGAGAATCGAAAGCA	CTGGACTGACGAAATCAAGGAA
*CR3*	CCATGACCTTCCAAGAGAATGC	ACCGGCTTGTGCTGTAGTC
*MARCO*	GGGTCAAAAAGGCGAATCTTTC	CCCTCTGGAGTAACCGAGCA
*TREM2*	CTGGAACCGTCACCATCACTC	CGAAACTCGATGACTCCTCGG
*ABCA-1*	GCTTGTTGGCCTCAGTTAAGG	GTAGCTCAGGCGTACAGAGAT
*APOE*	CTCCCAAGTCACACAAGAACTG	CCAGCTCCTTTTTGTAAGCCTTT
*PPAR-γ*	GGAAGACCACTCGCATTCCTT	GTAATCAGCAACCATTGGGTCA

### Western Blotting

Cell proteins were extracted using radioimmunoprecipitation assay (RIPA) lysis buffer (Beyotime, Shanghai, China) and quantified by the bicinchoninic acid (BCA) assay. The protein was isolated by sodium dodecyl sulfate–polyacrylamide gel electrophoresis (SDS-PAGE) and transferred to polyvinylidene difluoride (PVDF) membranes (EMD Millipore Corp., Billerica, MA, United States). Membranes were blocked with 5% skim milk in Tris-buffered saline with 0.1% Tween 20 (TBST) and incubated with primary antibodies, including anti-MARCO (1/1,000) and anti-β-actin (1/1,000). After being washed with TBST, membranes were then incubated with secondary antibody (1/10,000; Thermo Fisher Scientific, New York, United States), followed by incubation with chemiluminescent reagent (Millipore, United States) and detection using a Bio-Rad imaging system (Bio-Rad, Hercules, CA, United States). The protein bands were semiquantitatively analyzed by ImageJ software (National Institutes of Health, United States).

### Statistical Analysis

The results were statistically analyzed using GraphPad Prism software (version 7.00, La Jolla, CA, United States). All data are presented as the mean ± standard deviation (SD). Unpaired two-tailed Student’s *t*-test was used for two-group comparisons, and one-way or two-way ANOVA followed by Tukey’s *post hoc* test was performed for comparisons with more than two groups. *p*-Values less than 0.05 were considered statistically significant (in the figures, ^∗^*p* < 0.05, ^∗∗^*p* < 0.01, and ^∗∗∗^*p* < 0.001).

## Results

### Identification of Bone Marrow Mesenchymal Stem Cell-Derived Exosomes and Uptake Assay

**FIGURE 1:**
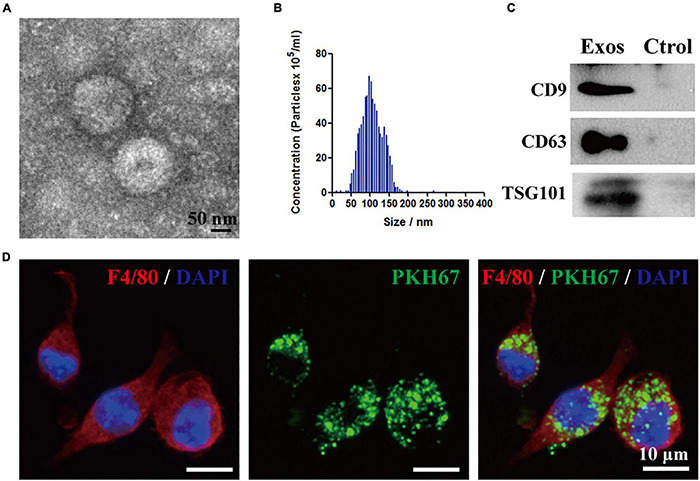
Identification of bone marrow mesenchymal stem cell-derived exosomes (BMSC-Exos) and BMSC-Exos uptake by macrophages *in vitro*. **(A)** Representative transmission electron microscopy (TEM) image of Exos. Scale bar = 50 nm. **(B)** Nanoparticle tracking analysis (NTA) of the Exos diameter and particle number. **(C)** Western blotting analysis of the exosome-specific markers CD63, CD9 and TSG101. **(D)** Representative immunofluorescence image of Exos (PKH67 labeled, green) uptake by macrophages (F4/80, red). Nuclei were counterstained with DAPI (blue). Scale bar = 10 μm. Exos, bone marrow mesenchymal stem cell-derived exosomes; Ctrol, control.

The isolated particles from the BMSCs were identified by TEM. Based on the TEM image in Figure 1A, BMSC-Exos exhibit a typical cup-shaped membrane vesicle morphology. The size distribution of the BMSC-Exos was defined by NTA with a diameter range from 50 to 150 nm (Figure 1B). Several proteins have been previously identified as exosome markers by Western blotting analysis, and the results showed that CD9, TSG101, and CD63 were expressed in BMSC-Exos (Figure 1C). To investigate the effect of BMSC-Exos on the phagocytosis capacity of macrophages, PKH67-labeled exosomes were cocultured with macrophages for 24 h. Fluorescence microscopy was used to detect exosome uptake by macrophages. Figure 1D demonstrates that exosomes could be taken up by macrophages.

### Bone Marrow Mesenchymal Stem Cell-Derived Exosome Administration Promoted Neurological Functional Recovery After Spinal Cord Injury

**FIGURE 2:**
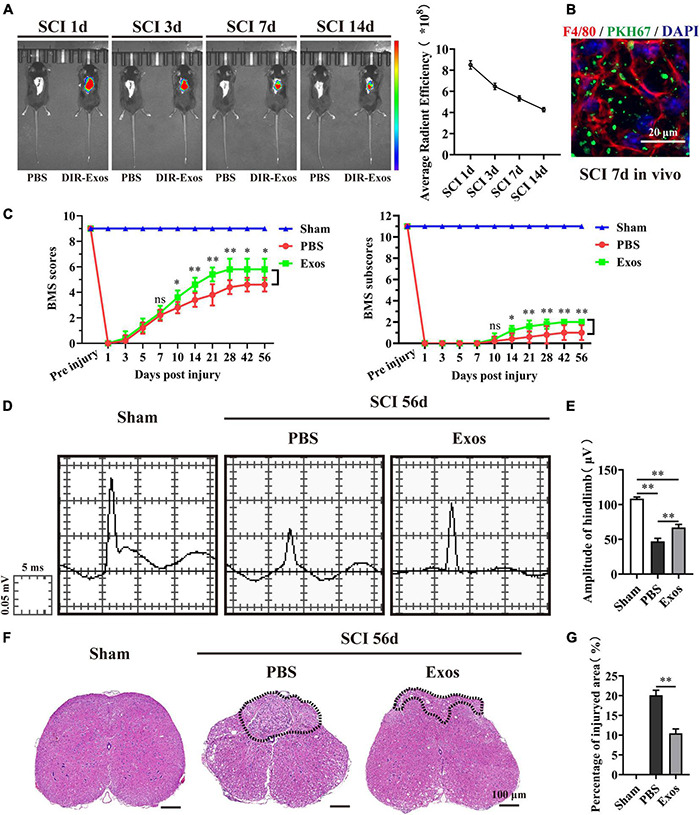
Bone marrow mesenchymal stem cell-derived exosomes (BMSC-Exos) improved neurological functional recovery and tissue repair after spinal cord injury. **(A,B)** *In vivo* tracing of DiR or PKH67-labeled exosomes (Exos) in the injured spinal cord. **(C)** Basso Mouse Scale (BMS) scores and subscores over time post-spinal cord injury (post-SCI) in the sham, phosphate-buffered saline (PBS), and Exos groups. **(D)** Representative images of motor evoked potentials (MEPs) in the sham, PBS, and Exos groups at 56 days post-SCI. **(E)** Quantification of **(C)** (*n* = 5 per group, values are the mean ± SD, ^∗^*p* < 0.05, ^∗∗^*p* < 0.01, one-way ANOVA). **(F)** H&E staining of injured spinal cords in the sham, PBS, and Exos groups at 56 days after SCI. The black dashed line outlines the injured sites. Scale bar = 100 μm. **(G)** Quantification of **(F)** (*n* = 5 per group, values are the mean ± SD, ^∗^*p* < 0.05, ^∗∗^*p* < 0.01, two-tailed Student’s *t*-tests).

In this study, to determine whether exosomes derived from BMSCs could exert beneficial effects on hindlimb motor function recovery after SCI, we first applied DiR or PKH67-labeled BMSC-Exos embedded in hydrogel for local injection on the injured surface of the spinal cord. We traced the distribution of the BMSC-Exos *in vivo* using the Xenogen IVIS Imaging System, and the image presented in Figure 2A indicates that the fluorescence signal of DiR-labeled BMSC-Exos was accumulated at the injury area of the spinal cord and could sustain until 14 days post administration. In contrast, no fluorescence signal was detected in the control mice, where PBS without DiR labeling was injected (Figure 2A). Furthermore, the immunofluorescence staining of spinal cord section revealed that the PKH67-labeled BMSC-Exos could be taken up by F4/80-positive macrophage cells at 7 days post-SCI (Figure 2B).

Then, the BMS scores and BMS subscores were used to assess the neurological functional recovery of mice treated with PBS and BMSC-Exos. As demonstrated in Figure 2C, we noticed that there was a significant increase in BMS scores (starting at 10 days post-SCI) and BMS subscores (starting at 10 days post-SCI) in mice treated with BMSC-Exos compared with mice treated with PBS and that these mice exhibited better motor functional improvement. To further evaluate the effect of BMSC-Exos on motor functional recovery, an electrophysiological test was applied. As presented in Figure 2D, the MEP amplitudes of hindlimbs were significantly higher in the BMSC-Exos-treated group than in the PBS group at day 56 postinjury, indicating that hindlimb motor function exhibited better recovery with administration of BMSC-Exos (Figure 2E). In addition, the traumatic lesion was clearly visible in the transverse view of the injured spinal cords (Figure 2F). Representative images of H&E staining are shown in Figure 2F, and these images confirmed that, following treatment with BMSC-Exos, the lesion site of the spinal cord was markedly smaller than that in the PBS-treated group (Figures 2F,G). Taken together, these results indicate that transplantation of BMSC-Exos has a neuroprotective effect on the injured spinal cord and could promote neurological functional recovery after SCI in mice.

### Bone Marrow Mesenchymal Stem Cell-Derived Exosomes Promote Macrophage Engulfment of Myelin Debris and Accelerate Axonal Growth After Spinal Cord Injury

**FIGURE 3:**
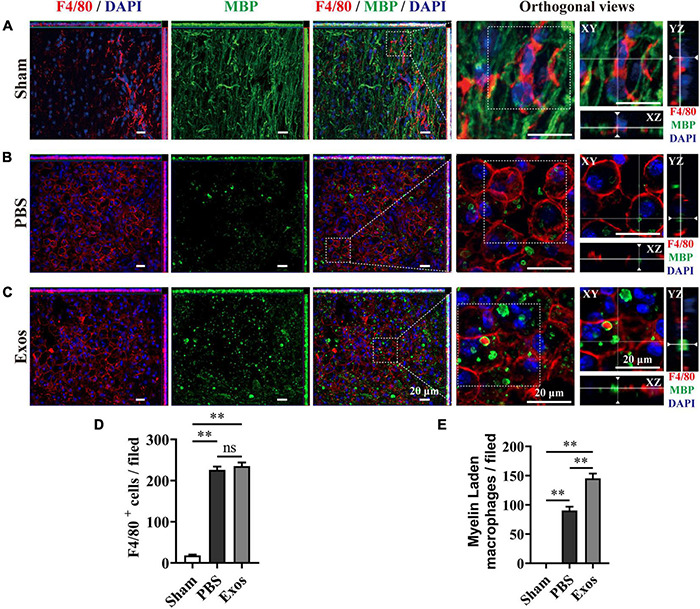
Bone marrow mesenchymal stem cell-derived exosomes (BMSC-Exos) promoted macrophage phagocytosis of myelin debris after spinal cord injury. **(A)** Representative immunofluorescence images of the macrophage marker F4/80 (red) and myelin marker myelin basic protein (MBP) (green) in the spinal cord tissue of the sham group. **(B,C)** Representative immunofluorescence images of myelin debris (MBP, green) uptake by macrophages (F4/80, red) in the lesion site of the spinal cord in the phosphate-buffered saline (PBS) and BMSC-Exos groups at 7 days post-spinal cord injury (post-SCI). Nuclei were counterstained with DAPI (blue). Scale bar = 20 μm. **(D,E)** Quantification of macrophages (F4/80, red) and myelin-laden macrophages (F4/80, red and MBP, green) in the lesion site at 7 days post-SCI (*n* = 5 per group, values are the mean ± SD, ns indicates no significance, ^∗∗^*p* < 0.01, one-way ANOVA).

**FIGURE 4:**
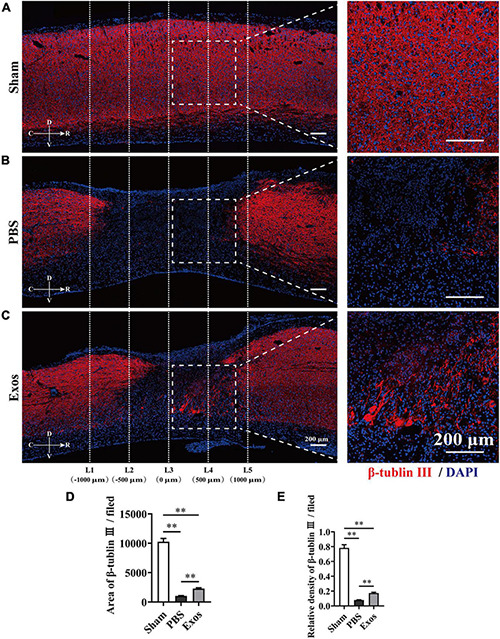
Bone marrow mesenchymal stem cell-derived exosomes (BMSC-Exos) improved axon growth after spinal cord injury *in vivo*. **(A–C)** Representative immunofluorescence images of the axon marker β-tubulin III (red) in the sham, phosphate-buffered saline (PBS), and Exos groups. Nuclei were counterstained with DAPI (blue). The white dashed line outlines the distance rostrally or caudally from the injured epicenter of the injured spinal cord. White dashed rectangular frames of region of interest (ROI) are selected for quantitative analysis of the axon area and density among different treatment groups. Scale bars = 200 μm. **(D,E)** Immunofluorescence semiquantification of the area and relative density of β-tubulin III (red) within the ROI in the spinal cord at 56 days post-spinal cord injury (post-SCI) (*n* = 5 per group, values are the mean ± SD, ^∗∗^*p* < 0.01, one-way ANOVA). ROI, region of interest.

To explore the underlying mechanism by which BMSC-Exos improve functional recovery after SCI, we assessed the effect of BMSC-Exos on promoting macrophage clearance of myelin debris. As presented in Figure 3A, the uninjured spine showed little detectable MBP. After SCI, myelin debris accumulated at the lesion site of the spinal cord. As a key phagocytic cell type, the infiltrating macrophages in the injured spinal cord evolved as part of the innate immune system and are responsible for engulfing myelin debris (Figures 3B–D). However, after injury, we showed that macrophage engulfment of myelin debris in the injured spinal cord was dysfunctional and that the macrophages failed to clear the debris (Figures 3B,D,E). Myelin debris clearance and removal are critical for axon remyelination. We applied BMSC-Exos for the treatment of SCI and tested whether BMSC-Exos could be useful for rescuing the impaired macrophage phagocytic capacity for myelin debris. Immunofluorescence staining revealed that BMSC-Exos-treated mice demonstrated a high level of phagocytosis in macrophage cells in the injured spinal cord, and a large amount of intracellular MBP-containing macrophage cells was observed when compared with the number in the PBS-treated group at 7 days post-SCI (Figures 3C–E). These *in vivo* data from SCI models indicate that macrophages engulf myelin debris after demyelination and that BMSC-Exos could promote the clearance of myelin debris by macrophages (Figures 3C–E). Regarding neuronal fiber staining, BMSC-Exos significantly promoted the expression of neuron-specific intermediate filaments based on β-tubulin III in the injured spinal cord compared with expression in the PBS groups. β-Tubulin III-positive fibers contained not only mature neuronal fibers but also some of the developing immature neuronal fibers. Rostrally from the injury epicenter of the spinal cord, the area and density of β-tubulin III-positive neuronal fibers in the BMSC-Exos groups were significantly higher than those in the PBS treatment groups at 56 days post-SCI (Figures 4A–E). These data indicated that BMSC-Exos enhances macrophage engulfment of myelin debris and may create a regenerative environment that is beneficial for axonal growth after SCI.

### Bone Marrow Mesenchymal Stem Cell-Derived Exosomes Promoted Macrophage Engulfment of Myelin Debris and Reduced Their Neurotoxicity

**FIGURE 5:**
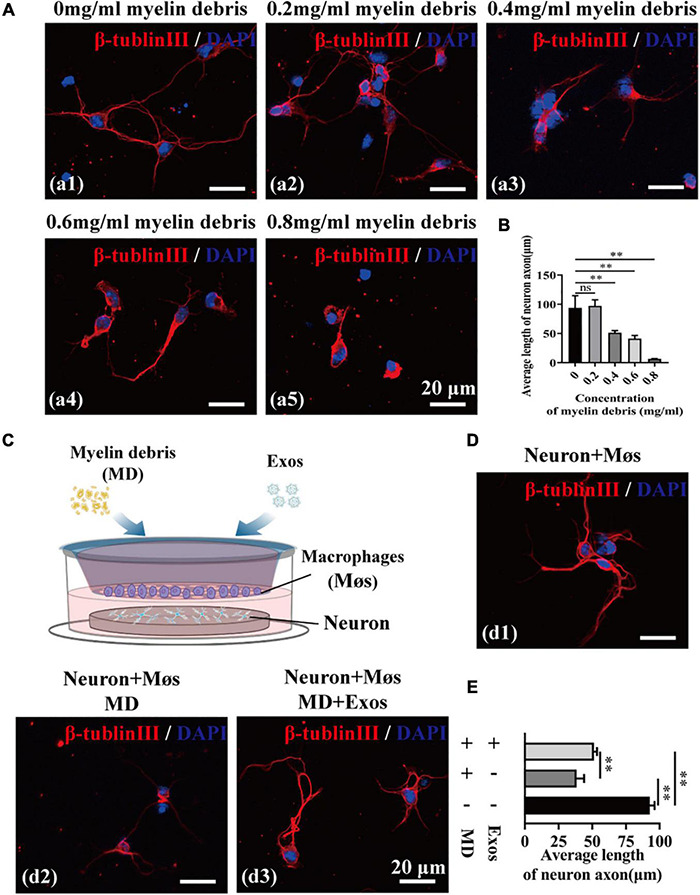
Bone marrow mesenchymal stem cell-derived exosomes (BMSC-Exos) promoted macrophage phagocytosis of myelin debris and reduced the neurotoxicity of myelin debris *in vitro*. **(A)** Representative immunofluorescence images of the neuron marker β-tubulin III (red) to reveal the neurotoxicity of myelin debris for inhibition of axon growth at different concentrations. Nuclei were counterstained with DAPI (blue). Scale bar = 20 μm. **(B)** Quantification of the axon length in **(A)** (*n* = 3 per group; values are the mean ± SD, ns indicates no significance, ^∗∗^*p* < 0.01, one-way ANOVA). **(C)** Schematic of the coculturing of myelin debris, macrophages, or BMSC-Exos with neurons. **(D)** Representative immunofluorescence images of neurons (β-tubulin III, red) under different coculture conditions. Nuclei were counterstained with DAPI (Blue). Scale bar = 20 μm. **(E)** Quantification of the axon length in **(D)** (*n* = 3 per group; values are the mean ± SD, ^∗∗^*p* < 0.01, one-way ANOVA). MD, myelin debris; Møs, macrophages.

We next investigated the neurotoxicity of myelin debris on axon regrowth using primary cortical neurons. After incubation with myelin debris, the axon length of cortical neurons was quantified by β-tubulin III staining. The results presented in Figure 5A reveal that myelin debris inhibited axonal regrowth of primary cortical neurons in a dose-dependent manner (Figures 5A,B). Myelin debris (0.8 mg/ml) significantly inhibited the axonal growth of cortical neurons (Figures 5A,B). Then, we cocultured myelin debris-treated cortical neurons with macrophages to promote the clearance of myelin debris by macrophages and to reduce the neurotoxicity of myelin debris on axon growth. As presented in Figures 5C–E, macrophage-alone treatment groups exhibited inefficient myelin debris engulfment and could not reduce neurotoxicity (Figures 5D(d2),E). However, compared with macrophages treated with myelin debris-loaded cortical neurons alone, BMSC-Exos-pretreated macrophages significantly reduced the neurotoxicity of myelin debris on axon growth (Figures 5D(d3),E).

**FIGURE 6:**
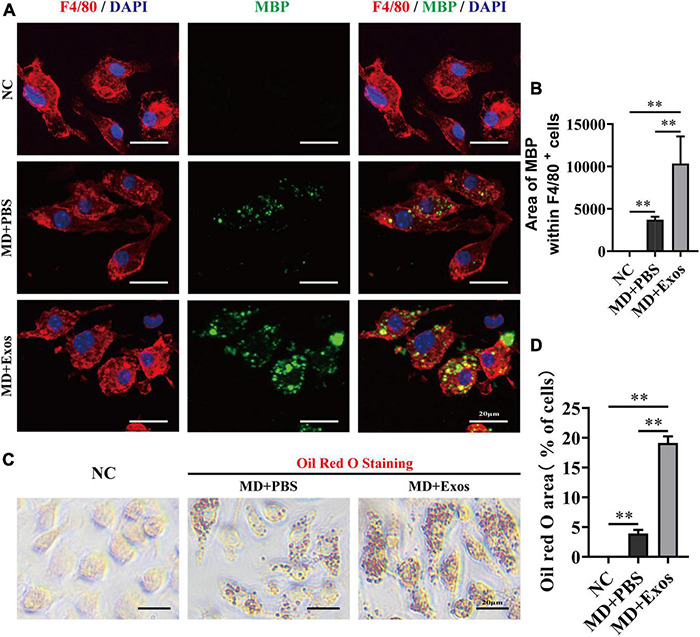
Bone marrow mesenchymal stem cell-derived exosomes (BMSC-Exos) promoted macrophage phagocytosis of myelin debris *in vitro*. **(A)** Representative immunofluorescence images of myelin debris [myelin basic protein (MBP), green] uptake by macrophages (F4/80, red) in the NC, MD + PBS, and MD + Exos groups. Nuclei were counterstained with DAPI (blue). Scale bar = 20 μm. **(B)** Immunofluorescence semiquantification of the area of MBP within the F4/80+ macrophages (*n* = 3 per group, values are the mean ± SD, ^∗∗^*p* < 0.01, one-way ANOVA). **(C)** Oil Red O staining of myelin debris uptake by macrophage from the NC, MD + PBS, and MD + Exos groups. Scale bars = 20 μm. **(D)** Quantification of Oil red O area with all cells in **(C)** (*n* = 3 per group; values are the mean ± SD, ^∗∗^*p* < 0.01, one-way ANOVA). MD, myelin debris; NC, negative control without added MD and Exos; PBS, phosphate-buffered saline.

We suspect that BMSC-Exos-treated macrophages may have a stronger phagocytic ability for clearing myelin debris than macrophage treatment alone, which may create a regenerative environment and directly promote axon growth. Visually, MBP immunofluorescent staining also confirmed that BMSC-Exos-treated macrophage cells contained significantly higher amounts of intracellular MBP than the amounts in PBS-treated cells at 24 h (Figures 6A,B). MBP-stained myelin debris was seen as scattered puncta around the cell nucleus and costained with F4/80 (Figure 6A). Oil Red O staining also revealed a large amount of intracellular lipids in each macrophage cell in the BMSC-Exos-treated group, indicating high levels of phagocytosis compared with levels in the PBS treatment groups (Figures 6C,D). These data indicated that myelin debris could be taken up by macrophages, and increased phagocytosis of myelin debris in macrophages was observed after administration of BMSC-Exos, which is consistent with the *in vivo* data from SCI model and is crucial for neurorepair and for aiding neurological functional recovery after SCI.

### Macrophage Receptor With Collagenous Structure Is Required for Myelin Debris Engulfment by Macrophages After Administration of Bone Marrow Mesenchymal Stem Cell-Derived Exosomes

**FIGURE 7:**
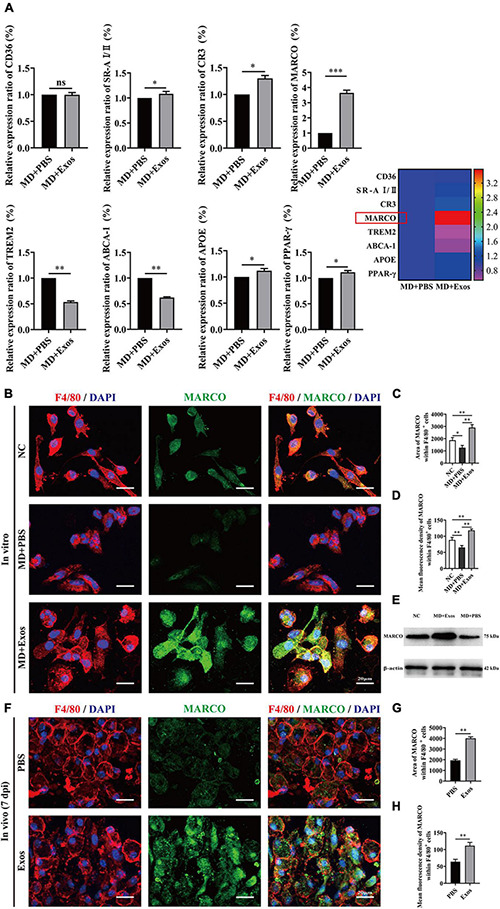
Bone marrow mesenchymal stem cell-derived exosomes (BMSC-Exos) upregulate the expression of macrophage receptor with collagenous structure (MARCO) in macrophages both *in vitro* and *in vivo*. **(A)** The relative gene expression and mapping of genes related to phagocytizing myelin debris of macrophages was detected by qRT-PCR (*n* = 3 per group, values are the mean ± SD, ns indicates no significance, ^∗^*p* < 0.05, ^∗∗^*p* < 0.01, ^∗∗∗^*p* < 0.001, two-tailed Student’s *t*-tests). **(B)** Representative immunofluorescence images of MARCO expression (MARCO, green) in macrophages (F4/80, red) in the NC, MD + PBS, and MD + Exos groups. Nuclei were counterstained with DAPI (blue). Scale bars = 20 μm. **(C,D)** Immunofluorescence semiquantification of the area and mean fluorescence density of MARCO within the F4/80+ macrophages cells (*n* = 3 per group, values are the mean ± SD, ^∗^*p* < 0.05, ^∗∗^*p* < 0.01, one-way ANOVA). **(E)** Western blotting analysis of MARCO expression in the NC, MD + Exos, and MD + PBS groups. **(F)** Representative immunofluorescence images of MARCO expression (MARCO, green) in macrophages (F4/80, red) in the PBS and Exos groups at 7 days post-spinal cord injury (post-SCI). Nuclei were counterstained with DAPI (blue). Scale bars = 20 μm. **(G,H)** Immunofluorescence semiquantification of the area and mean fluorescence density of MARCO within the F4/80+ macrophages cells (*n* = 5 per group, values are the mean ± SD, ^∗^*p* < 0.05, ^∗∗^*p* < 0.01, one-way ANOVA). MD, myelin debris; NC, negative control; 7 dpi, 7 days post-SCI; PBS, phosphate-buffered saline.

**FIGURE 8:**
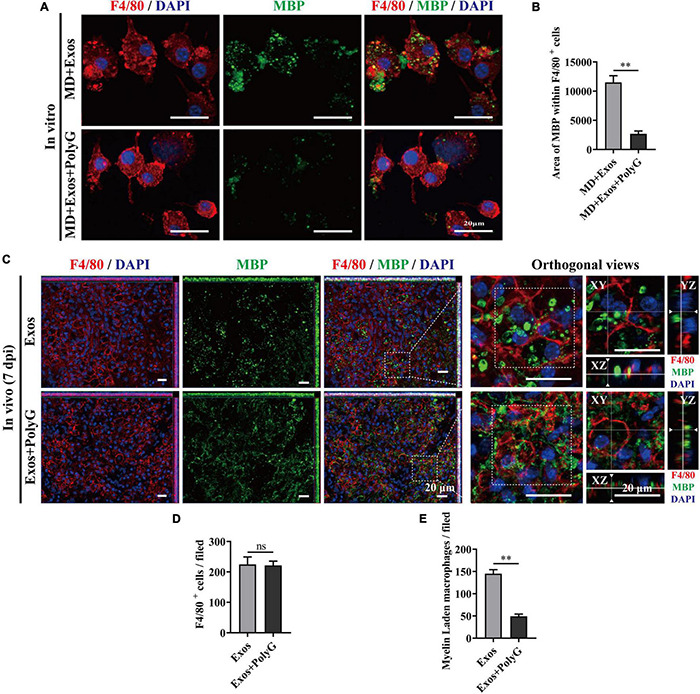
Blocking macrophage receptor with collagenous structure (MARCO) impaired the ability of bone marrow mesenchymal stem cell-derived exosomes (BMSC-Exos) to promote macrophage phagocytosis of myelin debris both *in vitro* and *in vivo*. **(A)** Representative immunofluorescence images of myelin debris [myelin basic protein (MBP), green] uptake by macrophages (F4/80, red) in the MD + Exos and MD + Exos + PolyG groups. Nuclei were counterstained with DAPI (blue). Scale bar = 20 μm. **(B)** Immunofluorescence semiquantification of the area of MBP within the F4/80+ macrophages (*n* = 3 per group, values are the mean ± SD, ^∗∗^*p* < 0.01, two-tailed Student’s *t*-tests). **(C)** Representative immunofluorescence images of myelin debris (MBP, green) uptake by macrophages (F4/80, red) in the lesion sites of the spinal cord in both the BMSC-Exos and Exos + PolyG groups at 7 days post-spinal cord injury (post-SCI). Nuclei were counterstained with DAPI (blue). Scale bar = 20 μm. (**D,E**) Quantification of macrophages (F4/80, red) and myelin-laden macrophages (F4/80 red and MBP green) per field in the lesion site at 7 days post-SCI (*n* = 5 per group, values are the mean ± SD, ^∗∗^*p* < 0.01, two-tailed Student’s *t*-tests). ns, not significant; MD, myelin debris; PolyG, MARCO antagonist; 7 dpi, 7 days post-SCI.

Macrophage phagocytosis could be mediated by multiple molecules. To understand the cellular and molecular mechanisms of macrophages after myelin debris engulfment following treatment with BMSC-Exos, we performed gene and protein level analyses to evaluate the role of BMSC-Exos in promoting myelin debris engulfment of macrophages. Several genes were upregulated in the BMSC-Exo treatment groups compared with the PBS treatment groups, including SR-AI/II, CR3, MARCO, APOE, and PPAR-γ. Genes including TREM2 and ABCA-1 were downregulated in the BMSC-Exo treatment groups compared with the PBS treatment groups (Figure 7A). Notably, among the upregulated genes, MARCO was the most markedly upregulated gene in macrophages after treatment with BMSC-Exos, which was confirmed by immunofluorescence data both *in vitro* (Figures 7B–D) and *in vivo* (Figures 7F–H) and Western blotting analysis in culture cells (Figure 7E). To evaluate the role of MARCO in modulating myelin debris engulfment by macrophages, we blocked MARCO in macrophages by using the MARCO antagonist PolyG and found that macrophage engulfment of myelin debris was significantly reduced or even abolished after adding PolyG (Figures 8A,B). Interestingly, myelin debris uptake by macrophages in injured spinal cord regions was also significantly inhibited (Figures 8C–E). Our *in vitro* and *in vivo* data both support that MARCO is required for myelin debris engulfment by macrophages after administration of BMSC-Exos.

### The Macrophage Receptor With Collagenous Structure Antagonist Attenuated the Effect of Bone Marrow Mesenchymal Stem Cell-Derived Exosomes on Promoting Neurological Functional Recovery After Spinal Cord Injury

**FIGURE 9:**
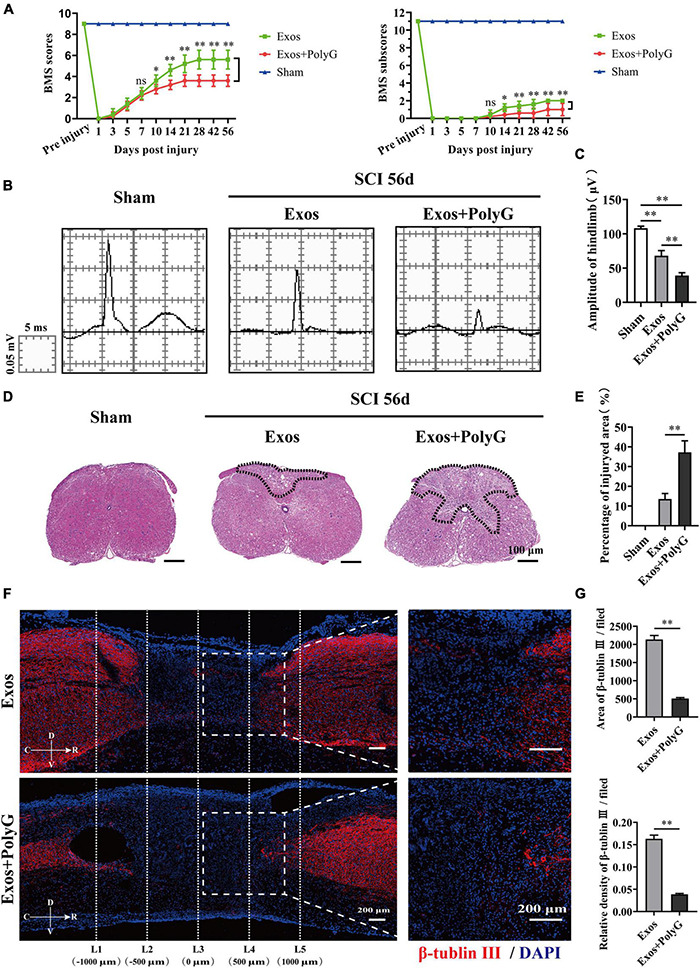
Blocking macrophage receptor with collagenous structure (MARCO) impaired the ability of bone marrow mesenchymal stem cell-derived exosomes (BMSC-Exos) to promote neurological functional recovery and tissue repair after spinal cord injury (SCI). **(A)** Basso Mouse Scale (BMS) scores and BMS subscores over time post-SCI in the sham, Exos, and Exos + PolyG groups. *n* = 5 per group. **(B)** Representative images of motor evoked potentials (MEPs) in the sham, Exos, and Exos + PolyG groups at 56 days post-SCI. **(C)** Quantification of amplitude of hindlimb in **(B)** (*n* = 5 per group, values are the mean ± SD, ^∗∗^*p* < 0.01, one-way ANOVA). **(D,E)** H&E staining and quantification of the lesion site in the sham, Exos, and Exos + PolyG groups at 56 days post-SCI. The black dashed line outlines the damage area. Scale bar = 100 μm (*n* = 5 per group, values are the mean ± SD, ^∗∗^*p* < 0.01, two-tailed Student’s *t*-tests). **(F)** Representative immunofluorescence images of the axon marker β-tubulin III (red) in the Exos and Exos + PolyG groups at 56 days post-SCI. Nuclei were counterstained with DAPI (blue). The white dashed line outlines the distance rostrally or caudally from the injured epicenter of the injured spinal cord. White dashed rectangular frames of ROI are selected for quantitative analysis of the axon area and density among different treatment groups. Scale bars = 200 μm. **(G)** Immunofluorescence semiquantification of the area and relative density of β-tubulin III (red) within the ROI in the spinal cord at 56 days post-SCI in **(F)** (*n* = 5 per group, values are the mean ± SD, ^∗^*p* < 0.05, ^∗∗^*p* < 0.01, one-way ANOVA). ROI, region of interest.

The MARCO antagonist PolyG attenuated the effect of BMSC-Exos on promoting macrophage clearance of myelin debris. This finding suggests that PolyG might inhibit neurological functional recovery after SCI. To test this hypothesis, we delivered PolyG mixed with hydrogel by local injection to the injured spinal cord surface of BMSC-Exos-treated mice. Then, we conducted behavioral assessment for an 8-week observation period to evaluate motor functional recovery using the BMS scoring systems. We detected obviously lower BMS scores (starting at 10 days post-SCI) and BMS subscores (starting at 10 days post-SCI) in the BMSC-Exos plus PolyG treatment groups than in the BMSC-Exos-treated alone mice (Figure 9A). We next performed electrophysiological test to evaluated functional motor regeneration after administration of PolyG. The electrophysiological analyses indicated that the MEP amplitude in the injured spinal cord in the BMSC-Exos plus PolyG combined treatment groups was obviously lower than that in the BMSC-Exos alone treatment groups (Figures 9B,C). Histological analyses at 1,000 μm rostrally from the injured epicenter demonstrated that animals receiving BMSC-Exos had a reduced lesion area at 56 days post-SCI, whereas PolyG exacerbated spinal cord lesions and attenuated the positive effect of BMSC-Exos on preserve the integrity of spinal cord (Figures 9D,E). In addition, the area and density of β-tubulin III-positive neuronal fibers in the BMSC-Exos + PolyG-treated SCI groups were significantly lower than those in the BMSC-Exo treatment groups at 56 days post-SCI (Figures 9F,G). These findings indicated that the MARCO antagonist PolyG attenuated the effect of BMSC-Exos on promoting neurological functional recovery after SCI.

## Discussion

**FIGURE 10:**
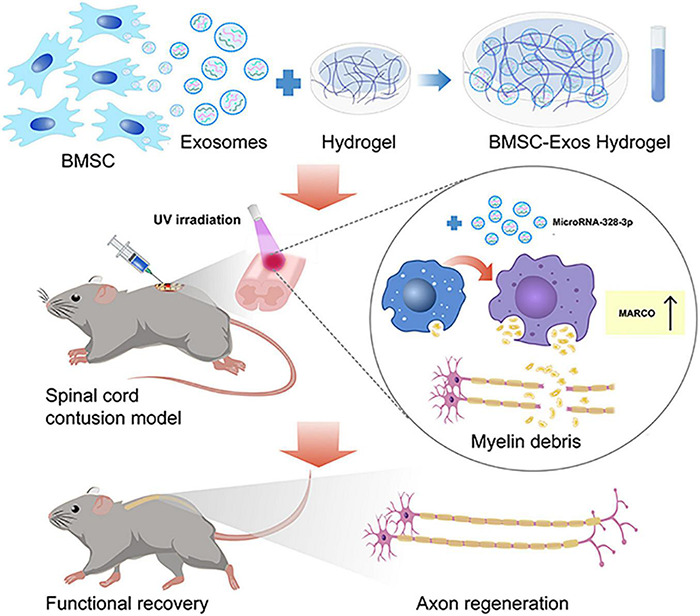
The schematic diagram depicts that the administration of bone marrow mesenchymal stem cell-derived exosomes (BMSC-Exos) mixed with hydrogel are transplanted to the surface of the injured spinal cord that could rescue normal macrophage function to clean myelin debris, creating a pro-regenerative environment that aided axon regeneration and neurological functional restoration after spinal cord injury (SCI). This process was mediated by upregulating the expression of macrophage receptor with collagenous structure (MARCO) in macrophages.

In the CNS, myelin debris generated after SCI contains neurite outgrowth inhibitors that prevent axonal regeneration and may aggravate neurological decline (Cignarella et al., 2020). Macrophages contribute predominantly to phagocytosis by processing myelin debris and may contribute to neurological function restoration after CNS injury (Van Broeckhoven et al., 2021). However, the phagocytic ability of macrophages is disturbed after SCI (Guo et al., 2016). Targeting the activation of macrophage phagocytosis function could become a promising therapy for SCI treatment (Gensel and Zhang, 2015; Guo et al., 2016). In our current study, we administered BMSC-Exos to rescue normal macrophage function, which resulted in a pro-regenerative environment that promoted axon regeneration and facilitated neurological functional restoration after SCI. Furthermore, our data suggest that BMSC-Exos upregulate the expression of MARCO in macrophages and increase the myelin debris content in macrophages (Figure 10). The inhibition of MARCO could impair the ability of exosomes to promote neurological functional recovery after SCI, indicating that exosomes enhance the phagocytosis ability of macrophages by regulating MARCO and providing a new therapeutic target for SCI treatment.

Acute spinal cord trauma damages the vasculature, leading to rupture of the blood–brain barrier, induces acute mechanical compression of the myelin sheath, generates myelin debris, and triggers a cascade of pathological processes, including immune cell infiltration and activation (Kopper and Gensel, 2018; Koganti et al., 2020). The sustained presence of myelin debris at the injury site of the spinal cord leads to neurological functional recovery failure because the accumulation of myelin debris containing inhibitor molecules resists axon regeneration (Cunha et al., 2020; Liu Y. et al., 2021). In addition, myelin debris could also activate the inflammatory response during SCI progression (Vargas et al., 2010; Zhou Q. et al., 2019; Wu et al., 2021). Therefore, clearance of myelin debris from the injury site is beneficial for neurological functional recovery after SCI.

Spinal cord injury repair requires the mobilization of immune cells to the injured site and the formation of a protective barrier that facilitates damaged myelin debris clearing and functional recovery (Imai et al., 2008; Nguyen et al., 2011; Wan et al., 2021). Multiple cell sources have been reported to have phagocytosis capacity (Wan et al., 2021). BMDMs are the major phagocytes that clear myelin debris in the CNS after injury (Zhou T. et al., 2019; Grajchen et al., 2020). Myelin debris can also be taken up by resident microglia (Lloyd et al., 2017; Cignarella et al., 2020; Berghoff et al., 2021). Our data demonstrated that myelin debris was taken up by F4/80-positive cells in the injured spinal cord, which has been used to define mature mouse macrophages and microglia at early stages. After SCI, microglia could be absent from the epicenter of the injured spinal cord (Cignarella et al., 2020). However, BMDMs, which are sensitive to microenvironmental changes and undergo either proinflammatory or anti-inflammatory polarization, start to accumulate in the injured spinal cord on day 3 and reach their peak at 7 days post-SCI (Gensel and Zhang, 2015; Milich et al., 2019). Thus, our observations indicate that a large percentage of F4/80+ cells may originating from BMDMs and take the responsible for myelin debris clearance in the injured spinal cord. Gene expression profiling also identified that BMDMs have a strong phagocytic response after SCI (Wang et al., 2015; Rolfe et al., 2017). Thus, we specifically focused on investigating the phagocytic properties of BMDMs in the spinal cord after injury.

Targeting the phagocytic capacity of macrophages could be a potential therapeutic strategy for SCI treatment. A few transcriptome analysis studies demonstrated that microglial cells could express high levels of certain specific genes, including Sall-1, Siglec-H, TMEM119, and P2RY12, which could be considered potential microglia-specific markers (Doring et al., 2015; Guo et al., 2016; Kucharova and Stallcup, 2017; Koganti et al., 2020). However, there is still no widely accepted cell marker to distinguish between BMDMs and microglia in the CNS, which needs further exploration.

As a cell-free treatment approach, there is high enthusiasm for the application of exosomes, which have potential use in immunotherapy to stimulate the immune system in regenerative medicine owing to their lowest immunogenicity and special biological properties (Zhang et al., 2019). BMSC-Exos have been identified to have unique composition profiles and exhibit functional roles in the regulation of immune responses (Zeng et al., 2020; Liu W. Z. et al., 2021). Multiple functional mechanisms of BMSC-Exos have a neuroprotective effect on SCI, which have been revealed. A recent study reported that BMSC-Exos can attenuate neuronal apoptosis and improve spinal cord functional recovery by activating autophagy in rats after SCI (Gu et al., 2020). Moreover, a study also demonstrated that the administration of BMSC-Exos could suppress the activation of A1 neurotoxic reactive astrocytes and inflammation after SCI (Liu et al., 2019). In our study, we report a novel mechanism of BMSC-Exos in SCI and demonstrated that innate immune cell macrophages are critical for SCI repair and that BMSC-Exo treatment could influence the phenotype of macrophages, enhance their phagocytic capacity for myelin debris clearance, and further improve motor functional restoration after SCI. Indeed, a previous study reported that in acute respiratory distress syndrome (ARDS), MSCs could transfer their mitochondria to immune macrophage cells and enhance macrophage phagocytic activity (Jackson et al., 2016). Unfortunately, the underlying molecular mechanisms of BMSC-Exos for enhancing phagocytic capacity of macrophage in our current study have not been investigated and need to be further determined. As natural carriers, exosomes exert their functions by transporting biological molecules, including proteins, DNA, mRNAs, and microRNAs, from parent cells to recipient cells and influence target cell function (Kalluri and LeBleu, 2020). Most importantly, what type of cargo BMSC-released exosomes deliver to targeted macrophage cells and the downstream signaling cascade that is activated in macrophage need to be further identified. Compared with those of conventional gene and drug carriers, such as liposomes, micelles, and different types of conjugates, exosomes are extracellular vesicles with a diameter of 40–200 nm that have many advantages, such as low toxicity, low immunogenicity, high stability in the circulation, and biological barrier permeability. These unique features make exosomes promising nanometer platforms or carriers for drug delivery (He et al., 2018; Kalluri and LeBleu, 2020; Hade et al., 2021; Samal et al., 2021). However, *in vivo* administration of unmodified exosomes demonstrated limited targeting ability, and the exosomes were normally trapped in nonspecific organs, such as the liver, spleen, kidney, and lung, and they showed rapid clearance from the blood circulation after systemic injection (Hade et al., 2021; Khan et al., 2021). To improve the targeting of exosomes and overcome the limitations of the systemic injection of exosomes, exosomes could be genetically modified, and direct targeting of inflated macrophages in the injured spinal cord should be considered.

Phagocytosis is a complex receptor-mediated process. Several phagocytic-related molecules could affect the phagocytic processes of macrophages, including CD36 scavenger receptor-A I/II (SR-AI/II), complement receptor 3 (CR3), MARCO, triggering receptor expressed on myeloid cells 2 (TREM2), ATP-binding cassette transporter A1 (ABCA-1), apolipoprotein E (APOE), and PPAR-γ (Van Broeckhoven et al., 2021). MARCO was first identified in the mouse, is an important phagocytic receptor, and belongs to the class A scavenger receptor molecules that have mostly been studied in cardiovascular disease (Savino et al., 2021). Human MARCO was found to be highly similar to mouse MARCO and was expressed on subsets of macrophages (Elomaa et al., 1998). In our study, we also found that the administration of BMSC-Exos could enhance macrophage phagocytosis activity for the engulfment of myelin debris and could promote axon growth and neurological functional healing after SCI, which was associated with a significant upregulation of the expression level of MARCO in macrophages. The pharmacological inhibition of MARCO using the MARCO antagonist PolyG may attenuate the effect of BMSC-Exos on macrophages, which may then display less phagocytic activity for myelin debris and may impair neurological healing in an *in vivo* SCI model. However, CD36 in macrophages showed no changes after treatment with BMSC-Exos. To our knowledge, CD36 is involved in lipid uptake by macrophages. Our results indicated that BMSC-Exos affect the expression of MARCO to mediate the phagocytic clearance of myelin debris by macrophages. This means that CD36 is not obligatory for macrophages to execute their major function during macrophage engulfment of myelin debris. Aside from the mentioned factor, another important factor is TREM2 (Cignarella et al., 2020). TREM2 is expressed by microglia and is a critical transcriptional regulator of microglial functions (Cignarella et al., 2020). TREM2 can promote microglial survival and mediate the phagocytic activity of microglia (Cignarella et al., 2020). TREM2 was downregulated in macrophages after administration of exosomes, which indicated that TREM2 may have the opposite effect on the macrophage clearance of myelin debris. Further research is needed to understand the role of TREM2 in regulating phagocytosis by macrophages after SCI. As a critical modulator that regulates phagocytosis functions in macrophages, targeting the key player in macrophages to increase myelin debris clearance could be a useful approach and beneficial for SCI repair.

In summary, our study indicates that BMDMs play a critical role in SCI repair and show phagocytic activity toward myelin debris and can create a regenerative environment for neurological functional recovery after SCI. However, the phagocytic capacity of macrophages is disrupted after SCI. BMSC-Exos could enhance the phagocytic function of macrophages by modulating MARCO expression and could promote macrophage engulfment of myelin debris, which would exert beneficial effects on axon regeneration and promote neurological functional recovery after SCI. BMSC-Exos could also serve as a cell-free immune therapy for modulating BMDM phagocytosis and have wide application prospects for SCI treatment.

## Data Availability Statement

The original contributions presented in the study are included in the article/supplementary material, further inquiries can be directed to the corresponding author/s.

## Ethics Statement

The animal study was reviewed and approved by Animal Care and Use Committee of Central South University.

## Author Contributions

JH, CD, and YC designed and supervised the study. XS, JZ, ML, and YX performed the experiments. YZ, JX, and RH analyzed the data. XS and YC wrote the manuscript. CD, JH, HL, TW, and YC revised the manuscript. All authors contributed to and approved the manuscript.

## Conflict of Interest

The authors declare that the research was conducted in the absence of any commercial or financial relationships that could be construed as a potential conflict of interest.

## Publisher’s Note

All claims expressed in this article are solely those of the authors and do not necessarily represent those of their affiliated organizations, or those of the publisher, the editors and the reviewers. Any product that may be evaluated in this article, or claim that may be made by its manufacturer, is not guaranteed or endorsed by the publisher.
